# The Clustered Regularly Interspaced Short Palindromic Repeats-Associated System and Its Relationship With Mobile Genetic Elements in *Klebsiella*

**DOI:** 10.3389/fmicb.2021.790673

**Published:** 2022-02-02

**Authors:** Yuqiao Zhou, Wei Zhou, Jinzhi Zhou, Jinchang Yan, Dingting Xu, Xiner Zheng, Shuaizhou Zong, Ping Jiang, Shiyi Tian, Jianzhong Han, Daofeng Qu

**Affiliations:** ^1^Food Safety Key Laboratory of Zhejiang Province, School of Food Science and Biotechnology, Zhejiang Gongshang University, Hangzhou, China; ^2^Zhejiang Provincial Center for Animal Disease Prevention and Control, Hangzhou, China; ^3^Agricultural and Rural Bureau of Wangdian Town, Jiaxing, China; ^4^The Second Affiliated Hospital, School of Medicine, Zhejiang University, Hangzhou, China; ^5^The Eighth People’s Hospital of Qingdao, Qingdao, China

**Keywords:** *Klebsiella*, CRISPR-Cas, type IV, plasmid, PCA

## Abstract

Microorganisms have developed many strategies in the process of long-term defense against external attacks, one of which is the clustered regularly interspaced short palindromic repeats (CRISPR)/CRISPR-associated proteins (Cas) bacterial immunological system. In this study, the whole genome of 300 strains of *Klebsiella* was collected, the CRISPR-Cas system in the strains was statistically analyzed, and the types and structures of CRISPR system in *Klebsiella* were explored, as well as the correlation between CRISPR and mobile genetic elements (MGEs). Through principal component analysis (PCA), we found that Cas gene, plasmids, integron, IS*1*, IS*609*, and enzymes of DNA metabolism were closely related to CRISPR-Cas. Compared the structural characteristics of plasmids, the DinG family helicases, Cas6, Csf2, and IS*5* were observed near the CRISPR loci in plasmid, which is also confirmed by the results of PCA that they may be important factors affecting the plasmid with CRISPR.

## Introduction

The genus *Klebsiella*, a member of the family *Enterobacteriaceae*, encompasses a huge diversity in terms of phylogenetic lineages, genomic content, pathogenic properties, and ecological distribution (Bridel et al., 2020). The occurrence of infectious antibiotic resistance in *Klebsiella* is a major problem worldwide. *Klebsiella pneumoniae* has a huge antibiotic resistance gene pool, which are shared with other *Enterobacteriaceae* mainly through self-transferring plasmids (Navon-Venezia et al., 2017). In genus *Klebsiella*, almost all modern antibiotic resistance (to carbapenems, cephalosporins, aminoglycosides, and now even colistin) are encoded in large (40–200 Kb) low-copy (16 per cell) conjugated plasmids (Samson et al., 2015).

Over the past decades, researchers have researched some CRISPR-Cas systems and Cas proteins in detail. CRISPR-Cas is an adaptive immune system which stores memories of encounters with foreign DNA which are mostly mobile genetic elements (MGEs) in unique spacing sequences extracted from the MGEs and inserted into the CRISPR array (Samson et al., 2015). Transcripts of CRISPR sequences are used to recognize homologous sequences and guide Cas nucleases to their unique targets when encountering familiar MGEs, resulting in inactivation of the latter (Barrangou and Horvath, 2017; Garcia-Martinez et al., 2018). Like all defense mechanisms, the CRISPR-Cas system evolved in a long arms race with MGEs, which has led to rapid evolution of some Cas gene sequences, primarily effect components, as well as significant diversity in the genetic composition of the CRISPR-Cas loci. CRISPR-Cas belong to nucleic acid-oriented defense systems, which is similar to eukaryotic RNA interference and the argonaute-centric defense mechanisms of prokaryotes (Takeuchi et al., 2012; Swarts et al., 2014; Koonin et al., 2017). Among these mechanisms, however, CRISPR-Cas has the complete ability to create immune memories, representing true adaptive immunity. The complexity and diversity of the CRISPR-Cas system implies a complex evolutionary history.

The CRISPR system is divided into two classes (polysubunit effector complex, mono-protein effector module) and six types (Types I, III, IV and Types II, V, VI), of which type I and type III are more studied (Ostria-Hernandez et al., 2015; Makarova et al., 2020). In the type I system, the CRISPR RNA (crRNA) complex recognizes the target DNA, which is then cleaved by Cas3. In type III system, the Cas10 protein is assembled into a complex that recognizes and cuts targets. The genomic CRISPR locus consists of three parts: *trans-*activated CRISPR RNA (tracrRNA) genes, Cas genes, and CRISPR repeat and spacers.

Some studies have identified type I-E and I-F CRISPR-Cas in many gram-negative *Enterobacteriaceae*, in addition, there are some studies have found that *K. pneumoniae* contains the CRISPR-Cas plasmid system which the CRISPR loci was on plasmid (Ostria-Hernandez et al., 2015; Medina-Aparicio et al., 2018; Qu et al., 2019). So far, there have been little research on type IV system located on mobile elements. The relationship between CRISPR-Cas and MGEs is very complicated. Some MGEs contributed to the origin and evolution of CRISPR-Cas, and conversely, the CRISPR-Cas system and its components were recruited by some MGEs (Faure et al., 2019).

This study investigated the diversity of the CRISPR-Cas system in *Klebsiella*, analyzed the relationship between MGEs and CRISPR-Cas system, especially the plasmid CRISPR-Cas. Plasmid CRISPR-Cas directed against other plasmids, it may provide another level of incompatibility in plasmid communities. Both plasmid and chromosomal CRISPR-Cas are evidently important determinants of the epidemiology of large antibiotic resistance plasmids in *Klebsiella*.

## Materials and Methods

### Strains Collection

All genome sequences of *Klebsiella* strains were retrieved and downloaded from National Center for Biotechnology information (NCBI) database.^1^ The whole genome download was saved as a FASTA format. Upload the whole genome sequence in the CRISPR Cas + + website^2^ to get each strain of CRISPR-Cas information (including the CRISPR locus, Cas gene, repetitive sequence and spacer, etc.). Only “confirmed” CRISPR loci were considered for further searching the presence of cas genes.

### Identification and Analysis of Clustered Regularly Interspaced Short Palindromic Repeats

Typical CRISPR repeats were sorted and stored in FASTA format, and ClustalX was used for multiple sequence alignment analysis (Thompson et al., 2002). The confirmed CRISPR loci were divided into seven categories according to the different repeat sequences, which were named as CRISPR1-7 (Makarova et al., 2011). Web logo^3^ was used to visualize the identified CRISPR site. These repeats were thought to be specific genetic markers for CRISPR. The secondary structures of single stranded RNA or DNA sequences were predited with RNAfold Web Server (Koonin, 2017).^4^ Current limits are 7,500 nt for partition function continuously and 10,000 nt for minimum free energy only predictions. MGEA7.0 software was used to construct phylogenetic trees of repeated sequences in *Klebsiella* CRISPR-Cas system for genetic evolution analysis.

### Spacer Sequence Analysis

In order to identify the spacing sequences matched with the mobile elements, the spacing sequences in the CRISPR loci were sorted and saved in FASTA format. The spacing sequences were BLASTN search in Genbank using standard BLASTN search, *e*-value < 10^–5^ homologous sequences with a 10% difference in sequence length, identifying the genetic moving element.

### Phylogenetic Analysis

Most Cas genes in *Klebsiella* belong to type I-E and type I-F. The corresponding Cas1 gene sequence was extracted from the whole genome sequence with BioEdit software and stored as FASTA file. The MGEA7.0 program was used to estimate nucleotides diversity and evolutionary distance, as well as to construct phylogenetic trees by using the Neighbor connection approach of the Juke-Cantor distance.

### The Distribution of Mobile Genetic Elements and Regulator in Strains

The FASTA file of the whole genome sequence obtained from NCBI was submitted to RAST website for gene annotation, and the obtained results were saved in the form of table. The genetic bioinformation of strains was obtained by NCBI database, and the number of insertion sequence, transposon, integron, and enzymes of DNA metabolism were counted. After all the data were integrated, the statistical correlation between the data and CRISPR was analyzed using principal component analysis (PCA).

### Plasmids Analysis

In order to analyze the characteristic structure of CRISPR-Cas system, the plasmids containing CRISPR-Cas were further analyzed. Download two CRISPR-Cas plasmid genome sequences from the NCBI database and upload them to RAST to annotate the genes sequence. The sequences were submitted to ISFinder, Integral, CRISPR-Target websites to supplement mobile elements and drug resistance gene information. Based on the above results, the diagrams of the plasmid structure were drawn (Ge et al., 2016). The correlation between plasmid CRISPR-Cas and MGEs was also analyzed by PCA, the steps were same as described above (Qu et al., 2019).

## Results

### Diversity of the Clustered Regularly Interspaced Short Palindromic Repeats-Associated Proteins System in *Klebsiella*

In April 2020, all 300 Klebsiella strains from April 2018 to March 2020 were downloaded from the NCBI database (Supplementary Table 1). The in-depth sequence analysis of the CRISPR-Cas system was implemented for genome sequences of 300 *Klebsiella* in the NCBI database. A total of 314 confirmed CRISPRs were identified in all genomes analyzed. These confirmed CRISPRs were distributed in 95 *Klebsiella* strains. Noticeably, 12 of the 95 confirmed CRISPR sites were found in plasmids.

**FIGURE 1:**
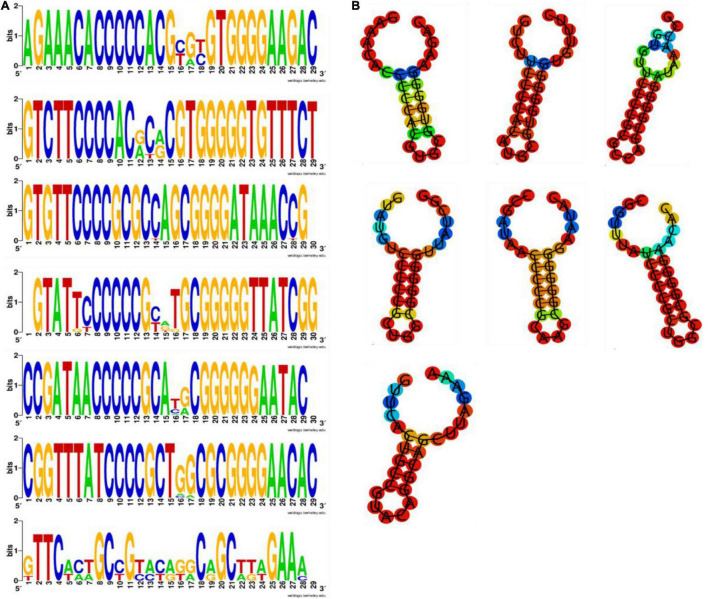
**(A)** The weblogo of repeats of CRISPR1∼CRISPR7. The sequence were first and terminal repeats of CRISPR1∼CRISPR7. **(B)** The secondary structure of repeats of CRISPR1∼CRISPR7. Secondary structure prediction of the most frequent sequence of the first and terminal repeats of each CRISPR was performed by RNAfold. The free energy of the thermodynamic ensemble was −11.60, −11.70, −15.20 −11.80, −12.70, −14.20, −8.60 kcal/mol.

The CRISPR loci were divided into seven groups according to the similarity of repeat sequences through multi-sequence alignment analysis, since the direct repeat length of CRISPR loci was similar within each locus by multiple sequence alignment analysis. The results showed that CRISPR2, CRISPR3, and CRISPR6 were the most common confirmed loci in all strains. The number of repetitions was 180, 147, and 60, respectively (Table 1). In order to better understand the features of these CRISPR groups, Weblogo was used to analyze the differences between repeats. The results suggest that CRISPR2 and CRISPR3 have fewer mutations and higher frequencies (Figure 1A). Previous researches have indicated that CRISPR repeats may form stable hairpin-like secondary structures (classical stem-loop) due to the partially palindromic nature, which contains a large and a small loop at both ends of each repeats of CRISPR (Victor et al., 2007; Bhaya et al., 2011). The Figure 1B showed that seven CRISPR groups have two loop at each end of the RNA secondary structure and a stem in the middle, which is 5–7 nucleotides in length and highly conserved. Some of the clusters present stable, highly conserved RNA secondary structures. Stable secondary structures exhibit multiple compensatory base changes in the stem region, which indicated evolutionary and functional conservation (Victor et al., 2007). The results showed that CRISPR2 and CRISPR3 have the lowest minimum free energy (MFE), meaning they have the most stable RNA secondary structure.

**TABLE 1:** The information of the confirmed CRISPR1∼CRISPR7.

CRISPR	Type	Repeat sequence (5′–3′)	No. of strains	No. of repeats	Frequency (%)
	Typical repeat	GAAACACCCCCACGTGCGTGGGGAAGAC	10	127	57.21
	Repeat variants	AGAAACACCCCCACGTGCGTGGGGAAGAC		12	5.41
		AGAAACACCCCCACGCGTGTGGGGAAGAC		20	9.01
CRISPR1		AGAAACACCCCCACGCATGTGGGGAAGAC		4	1.80
		AGAAACACCCCCACGCGTGTGGGGAAGA		10	4.50
	Terminal repeat	GAAACACCCCCACGCGTGTGGGGAAGAC		25	11.26
		GAAACACCCCCACGCATGTGGGGAAGAC		24	10.81
	Typical repeat	GTCTTCCCCACGCACGTGGGGGTGTTTC	38	405	44.90
	Repeat variants	GTCTTCCCCACACGCGTGGGGGTGTTTCT		166	18.40
		GTCTTCCCCACGCACGTGGGGGTGTTTCT		149	16.52
		GTCTTCCCCACACGCGTGGGGTGTTTCT		7	0.78
CRISPR2		TCCCCACACGCGTGGGGGTGTTTCT		10	1.11
	Terminal repeat	GTCTTCCCCACACACGTGGGGGTGTTTC		12	1.33
		GTCTTCCCCACATGCGTGGGGGTGTTTC		78	8.65
		GTCTTCCCCACACGCGTGGGGGTGTTTC		57	6.32
		GTCTTCCCCACGTGCGTGGGGGTGTTTC		18	2.00
	Typical repeat	GTGTTCCCCGCGCCAGCGGGGATAAACCG	17	612	97.45
CRISPR3	Repeat variants	GTGTTCCCCGCGCTAGCGGGGATAAACCG		6	0.96
	Terminal repeat	GTGTTCCCCGCGCCAGCGGGGATAAACTGG		10	1.59
	Typical repeat	GTATTCCCCCCGCATGCGGGGGTTATCGG	13	79	36.74
	Repeat variants	AGTATTCCCCCCGTGTGCGGGGGTTATCGG		5	2.33
	Terminal repeat	CCCCCCGCATGCGGGGGTTATCGG		19	8.84
CRISPR4		GTATGTCCCCCGCTGGCGGGGGTTATCGG		12	5.58
		GTATTCCCCCGCTTGCGGGGGTTATCGG		29	13.49
		GTATTCCCCCCGTGTGCGGGGGTTATCGG		71	33.02
	Typical repeat	CCGATAACCCCCGCATGCGGGGGGAATAC	11	70	45.45
	Repeat variants	CGATAACCCCCGCATGCGGGGGGAATAC		10	6.49
	Terminal repeat	CCGATAACCCCCGCACACGGGGG		43	27.92
CRISPR5		CCGATAACCCCCGCATGCGGGGGGAATACT		9	5.84
		CCGATAACCCCCGCATGCGGGGG		11	7.14
		CCGATAACCCCCGCAAGCGGGGGGAATAC		11	7.14
	Typical repeat	CGGTTTATCCCCGCTGGCGCGGGGAACAC	12	354	93.65
CRISPR6	Repeat variants	GGTTTATCCCCGCTGGCGCGGGGAACAC		7	1.85
	Terminal repeat	CGGTTTATCCCCGCTCACGCGGGGAACAC		7	1.85
		CGGTTTATCCCCGCTAGCGCGGGGAACAC		10	2.65
	Typical repeat	GTTCACTGCCGTACAGGCAGCTTAGAAA	7	44	55.00
	Repeat variants	TAAGCTGCCTGTACGGCAGTGAA		4	5.00
	Terminal repeat	TTTCTAAGCTGCCTGTACGGCAGTGAACA		4	5.00
		ATTCGGCTTGAGAGCCGTTTCCA		4	5.00
		GGGAATAAGTCACTGAAAGTAA		4	5.00
CRISPR7		GGAAACGGCACAAAAGCCGAATCGAAGCAAGTTACTGAAAATAA		4	5.00
		GGAATCGGCGCAAAAGCCGAATCGAAGCAAGTTACTGAAAATAA		4	5.00
		GCATTCGTCCCAAGAGCCGAA		4	5.00
		GGGAATAAGGCATTGAAAGTTA		4	5.00
		GCATTCGTCCCAAGAGCCGAT		4	5.00

### The Effect of Spacer Structure on Clustered Regularly Interspaced Short Palindromic Repeats Loci

**FIGURE 2:**
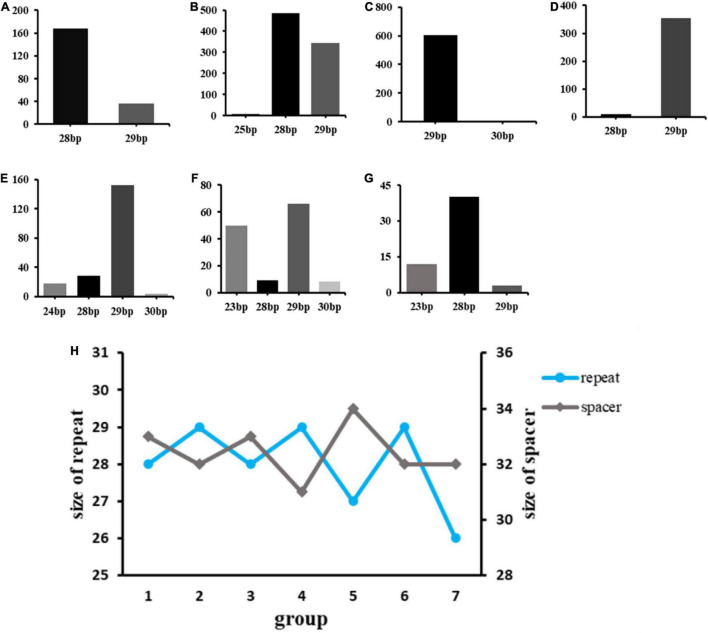
Six Groups of CRISPR spacer size variability. The relationship between the size of repeat and spacer among six groups: **(A)** Group 1 spacers; **(B)** Group 2 spacers; **(C)** Group 3 spacers; **(D)** Group 4 spacers; **(E)** Group 5 spacers; **(F)** Group 6 spacers; **(G)** Group 7 spacers; **(H)** The x-axis represents the size of the CRISPR spacers, the y-axis represents the number of the CRISPR spacer. The size of repeat and spacer were inversely correlated.

According to data statistics, the total number of spacers in *Klebsiella* strains was 2549. The analysis on the number and length of spacer in CRISPR (Figures 2A–H). The spacer size distribution indicated that variability was greatest in Group 3 (Figure 2C), and Group 6 (Figure 2F) had the lowest variability (*P* < 0.05). The data implied a negative correlation between the size of the repeat and spacer (Figure 2H).

From the perspective of base matching with exogenous gene sequences, we found that CRISPR 1–7 had 51, 197, 111, 176, 100, 68, and 8 special spacing sequences, respectively, and the exogenous matched sequences were 262, 1512, 1617, 1040, 751, 1197, and 73, respectively. Most of these foreign sequences come from insertion sequences (IS), transposons, plasmids and phages, which confirmed the mechanism of spacer formation. The presence of spacers was matched with elements associated with antibiotic resistance gene mobilization (e.g., IS5, Tn3). Taken together, the current findings confirm that repeated sequences are negatively correlated with the size of the spacer block. Prokaryotes memorize invader information by incorporating alien DNA as spacers into CRISPR arrays, and it may alter the function of CRISPR-Cas system, while PCR experiments and sequencing can be performed in the future.

### Distribution and Structural Feature of Clustered Regularly Interspaced Short Palindromic Repeats-Associated Proteins Genes in Strains

Clustered regularly interspaced short palindromic repeats-associated protein is an important component of CRISPR-Cas immune system function and an indicator of immune system activity. In this section, we found that there were 29 strains that have cas genes in the order of Cas3-Cse1-Cse2-Cas6-Cas7-Cas5-Cas1-Cas2, and there were 10 strains have the *cas* genes in the order of Cas2-Cas1-Cas5-Cas7-Cas6-Cse2-Cse1-Cas3, 10 strains have the *cas* genes in the order of Cas3-Cse1-Cse2-Cas7-Cas5-Cas6-Cas1-Cas2, 8 strains have the *cas* genes in the order of Cas2-Cas1-Cas6-Cas5-Cas7-Cse2-Cse1-Cas3 and 1 strain has the *cas* genes in the order of Cas1-Cas3-Cas 2-Csy1-Csy2-Csy3-Cas 6, 1 strain has the *cas* genes in the order of Cas 3-Cse1-Cas 6-Cas 5-Cas 1-Cas 2.

**FIGURE 3:**
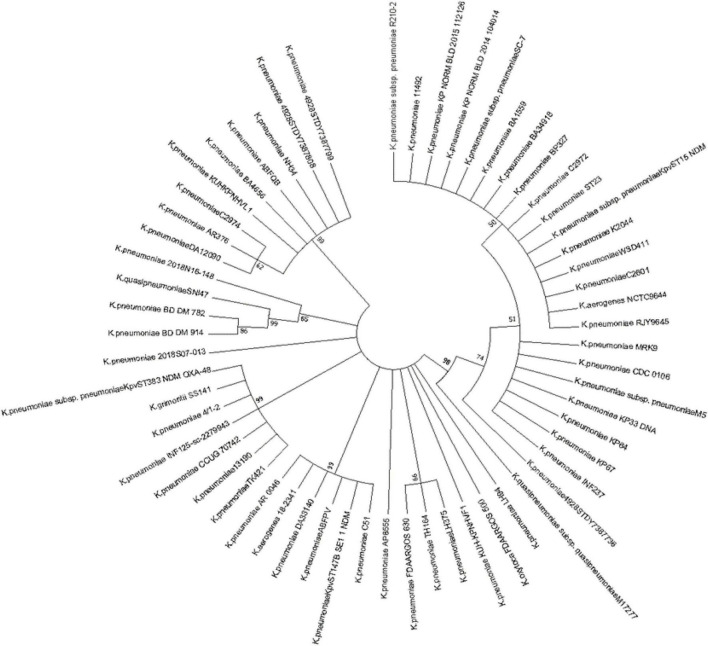
The evolutionary tree of Cas1 of all strains. The Cas1 has 57 strains, respectively. The Cas1 genes sequence were obtained by searching for the complete genome sequences in Genbank. Strains in one branch indicate most evolutionary similarities, the branch represented that these sequences could be divides into groups by certain values and the percentage of each branch showed the sequence similarity, and the evolutionary distance scale of Cas1 in 0.10.

One important finding was that in almost every type, the Cas1 gene is always present in the same site therefore its reasonable to assume the cas1 gene is ubiquitous in *Klebsiella*’s CRISPR-Cas system (Figure 3). By constructing homologous evolutionary trees, Cas1 gene of different strains was compared to conduct further research and analyze the role of Cas1 gene in *Klebsiella* evolution. Thus, Cas1 gene can be used to roughly classify bacteria among species according to nucleotide similarity. Compared to other Cas genes, Cas1 is more representative, because almost all bacteria contain Cas1.

### The Relationship Between Clustered Regularly Interspaced Short Palindromic Repeats and Mobile Genetic Elements, Regulators, and Enzymes of DNA Metabolism

Clustered Regularly Interspaced Short Palindromic Repeats-Associated Proteins systems are known to resist MGE invasions, such as plasmids, phages, and integrative conjugative element (ICE) which often carry antibiotic resistance genes (ARGs). The relationship between CRISPR-Cas system and MGEs is complex and diverse. MGEs can promote the high variation of CRISPR loci in bacteria, and CRISPR can defend against MGE attacks. There are studies have shown that some strains lacked CRISPR and these strains possess significantly more phages and plasmids than CRISPR harboring strains (Qu et al., 2019). Meanwhile, enzymes of DNA metabolism played a crucial role in the transcription and translation of CRISPR systems.

**FIGURE 4:**
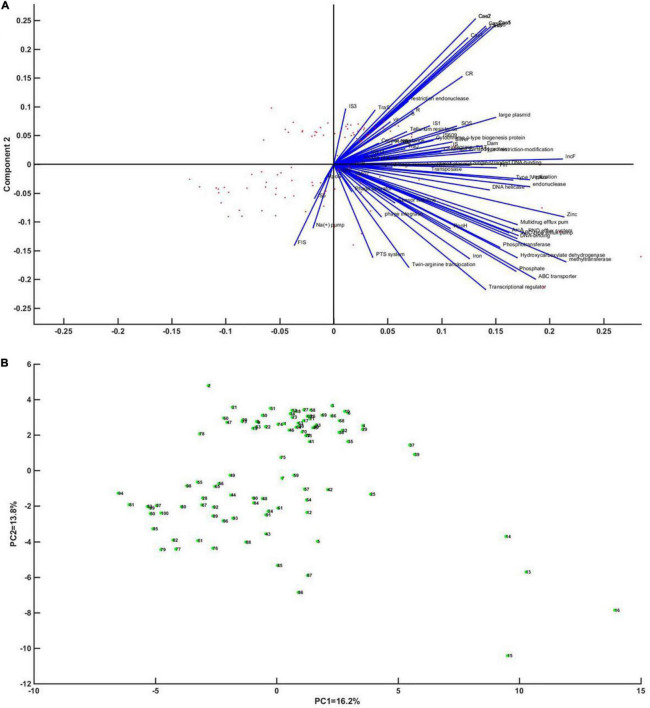
Principal component analysis of CRISPR of *Klebsiella* in relation to mobile genetic elements, regulators and enzymes of DNA metabolism. **(A)** Correlation vector diagram of each factor of principal component axis (blue line) and *Klebsiella* strains (red dot). A vector represents the correlation of each factor with the axis of the first principal component. **(B)** An enlarged view of the point representing *Klebsiella*.

Principal component analysis was performed on whether CRISPR has an impact on genetic mobile elements, regulators, and enzymes of DNA metabolism. Through analysis, it was found that Cas gene was most closely related to CRISPR (the coefficients were 56–62%), and plasmids, integrons, IS*1*, IS*609*, and enzymes of DNA metabolism had high correlation with CRISPR (the coefficients were 29.4, 26.6, 20.4, 42.1, and 35.4%, respectively) (Figure 4).

### The Effect of Clustered Regularly Interspaced Short Palindromic Repeats-Associated Proteins System on Plasmids

**FIGURE 5:**
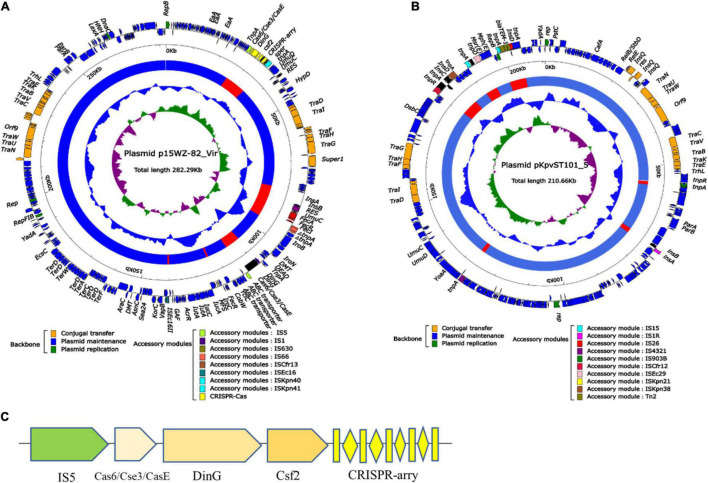
**(A)** Schematic diagram of the Plasmid p15WZ-82_Vir. **(B)** Schematic diagram of the Plasmid pKpvST101_5. According to the function of the gene classification, use arrows to represent the gene, and color. **(C)** Local structure of plasmid CRISPR.

To observe the structural characteristics of the CRISPR-Cas system, the plasmid containing CRISPR (p15WZ-82_Vir) and the plasmid without CRISPR (pKpvST101_5) were analyzed, the distribution of mobile genetic elements and regulatory factors on the two plasmids were compared (Figure 5). After comparing the graphical results of the two plasmids, it was found that both plasmids contained a comparable number of *Tra* family genes, IS sequences, transposons, and integrons, indicating the diversity levels of the two plasmids were similar. Interestingly, we also found that the CRISPR sites on the P15WZ-82_VIR plasmid contained DinG family helicase, type I-E CRISPR-associated protein Cas6/Cse3/CasE, CRISPR-associated protein Csf2 and IS*5* (Figure 5C). These genes and mobile elements were not found in pKpvST101_5, the plasmid that does not contain the CRISPR-Cas system. Therefore, we speculate that these genes and mobile elements may be important factors affecting the generation or evolution of CRISPR on the plasmid.

### The Effect of Mobile Genetic Elements on Clustered Regularly Interspaced Short Palindromic Repeats-Associated Proteins System of Plasmids

**FIGURE 6:**
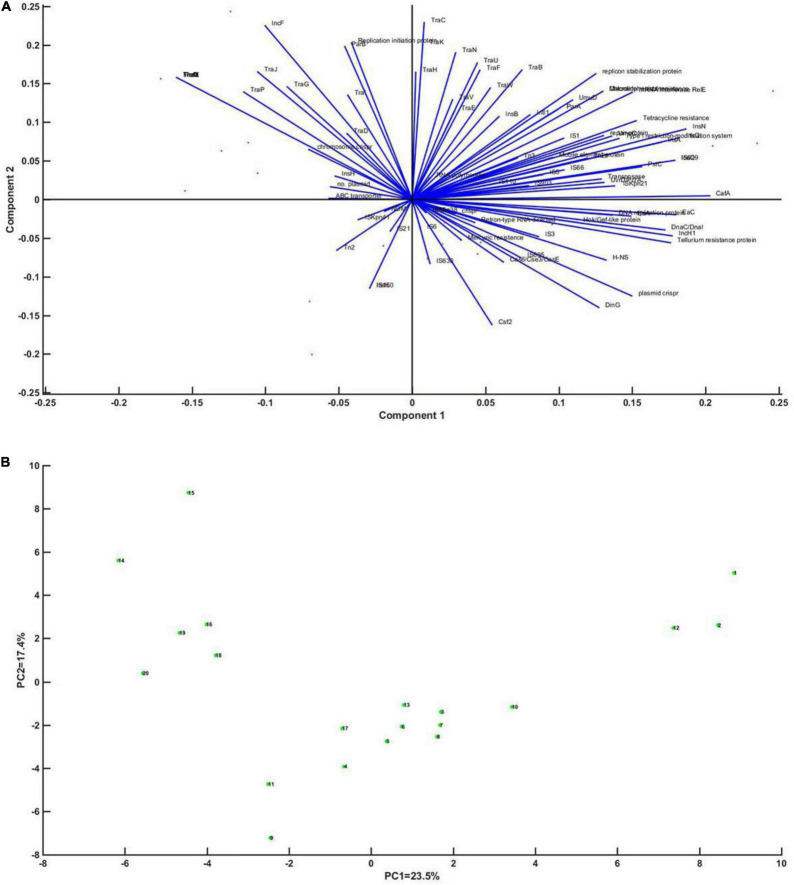
Principal component analysis of the CRISPR plasmid of *Klebsiella* with mobile genetic elements, regulatory factors, and enzymes of DNA metabolism. **(A)** Correlation vector diagram of principal component axis (blue line) and each factor of *Klebsiella* strain (red dot). The vector represents the correlation of each factor to the axis of the first principal component. Panel **(B)** represents an enlarged view of *Klebsiella*’s point.

In order to comprehensively analyze the effect of MGEs on the emergence and development of CRISPR plasmids, the whole gene sequences of 12 CRISPR-containing and 8 CRISPR-free plasmids were collected (Supplementary Table 2). The 20 plasmids were annotated, and principal component analysis was performed on the annotated results. Sequence alignment of 12 plasmids showed that the similarity among the 12 plasmids was not high (Supplementary Table 3). PCA results showed that DinG family helicase, Cas6/Cse3/CasE, Csf2, IS5, and plasmid CRISPR had relatively high coefficients (81.2, 53.3, 73.8, 47.1%, respectively) (Figure 6).

## Discussion

In this paper, the distribution, type and spacer sequence of CRISPR-Cas system in *Klebsiella* were researched. Among the collected strains, about one third contained CRISPR-Cas system, and most of the CRISPR-Cas system belonged to type I-E. twelve strains were found to have CRISPR gene loci in plasmids.

The CRISPR-Cas system provides bacteria with adaptive immunity against plasmids and other MGEs. In *Klebsiella*, the plasmid specific spacer obtained from the CRISPR spacer of chromosomes can provide immunity to the plasmid for the strain. Huang et al. (2017) proposed in the study of multi-drug resistant *K.* pneumoniae that drug-resistant genes could be integrated from the plasmid to the chromosome by using the CRISPR-Cas system. Muhammad and Jonathan also concluded that obtaining new spacer sequences in the CRISPR-Cas array could induce the degradation of its targeted plasmids in the host, prompting the transfer of drug-resistant genes on the plasmids to chromosomes or other related mobile genetic elements under the pressure of antimicrobial selection (Kamruzzaman and Iredell, 2019). Many strains carry multiple plasmids, the acquisition of new plasmids reduces the growth rate and fitness of the plasmid-carrying host, thus placing a burden on the host. Obtaining plasmid-mediated CRISPR spacers targeting other plasmids and host chromosomes may facilitate the collaborative integration of plasmids with each other or into host chromosomes, thereby improving the stability and compatibility of plasmids (Kamruzzaman and Iredell, 2019).

The type-IV CRISPR Cas system is equivalent to a simplified version of the type-I CRISPR Cas system, with a genetic makeup similar to that of type-I. However, the Cas protein sequences of type-IV systems are quite different from those of other type-I systems, so they are classified as different systems (Makarova et al., 2015). Type-IV system has two variants (subtype IV-A and subtype IV-B), both of which contain highly differentiated effector module genes of Cas5 (Csf3), Cas7 (Csf2), and Cas8-like large subunit (Csf1), but subtype IV-A also encodes the DinG family helicase. The presence of DinG helicase (csf4), only previously reported in type-IV CRISPR-Cas (Koonin et al., 2017; Pinilla-Redondo et al., 2020). All of the complete genomes that characterize the type-IV CRISPR Cas system are encoded by bacterial plasmids, bacteriophages, or other uncharacterized integrated elements (Faure et al., 2019). In addition, some type-IV CRISPR Cas loci encode predictive enzymes of ADP Ribosyl transferase family (ART), including bacterial toxins. Together with the type-IV system’s Cas proteins, these enzymes may help suppress the host CRISPR Cas or other defense systems, ensuring the stability of plasmids and prophages (Shabbir et al., 2016).

The type-IV CRISPR-Cas system on plasmids lacks target enzymes (Cas3 or Cas10 genes). In the study of Muhammad Kamruzzaman and Jonathan R. Iredell, it was mentioned that the positive plasmids of the CRISPR-Cas system of type-IV *Klebsiella* pneumococci were only found in the bacteria of type I-E chromosome CRISPR-Cas, which made up for the lack of target cutting function in the CRISPR plasmids, considering that there may be a cross between the plasmid and the CRISPR chromosome (Kamruzzaman and Iredell, 2019). In this study, we found that some strains only contained the type-IV CRISPR-Cas system, which may be the result of the continuous evolution of CRISPR under environmental pressure and certain MGEs. The impact of MGEs on the CRISPR Cas system occurs in many independent situations, including the ability to eliminate interference. However, the actual effect of the derived CRISPR-Cas system on plasmids remains to be discovered.

The CRISPR-Cas system was found not only in plasmids, but also in other MGEs. Including phages, Tn7 transposition elements and integrative conjugative elements (Koonin et al., 2020). Recruitment of CRISPR-Cas defense systems by different MGEs may have contributed to the evolution of MGEs and defense systems. Some CRISPR adaptation modules (e.g., Cas1, Cas2, Cas4, etc.) are thought to have evolved from different transposons. Transposon is an extensive MGE that can be reproduced by recombinases that insert elements into new locations in the host genome, and involved in DNA replication, DNA repair, and sometimes reverse transcription (Faure et al., 2019). Most of the CRISPR-Cas carried by MGEs only retained some of their original functions, and the CRISPR-Cas system was preserved in the evolution of MGEs by inhibiting the host defense to gain an advantage in the conflict with MGEs. There is a complex functional and evolutionary relationship between CRISPR-Cas and MGEs, including the similarity between CRISPR-Cas function and the various nuclease reactions in the life cycle of MGEs (Faure et al., 2019). Much of the biological information involved needs further exploration and discovery.

## Conclusion

This study focuses on the CRISPR-Cas system in *Klebsiella* to explore various factors affecting CRISPR and the relationship between CRISPR and mobile genetic elements. The analysis shows that CRISPR interferes with and protects against foreign mobile devices, while some genes and mobile genetic elements may also have significant influence on the emergence and evolution of CRISPR. Explored various types of CRISPR-Cas systems in *Klebsiella*, which is prevalent worldwide, it’s of great significance to research the plasmid - mediated resistance transmission of *Klebsiella* in the future.

## Data Availability Statement

The original contributions presented in the study are included in the article/Supplementary Material, further inquiries can be directed to the corresponding author.

## Author Contributions

DQ conceived the project. YZ, WZ, JZ, and JY participated in its design. YZ, WZ, PJ, XZ, SZ, and ST performed the data mining and analyses. YZ, DQ, and DX wrote and revised the manuscript with input from all authors. The author(s) read and approved the final manuscript.

## Conflict of Interest

The authors declare that the research was conducted in the absence of any commercial or financial relationships that could be construed as a potential conflict of interest.

## Publisher’s Note

All claims expressed in this article are solely those of the authors and do not necessarily represent those of their affiliated organizations, or those of the publisher, the editors and the reviewers. Any product that may be evaluated in this article, or claim that may be made by its manufacturer, is not guaranteed or endorsed by the publisher.

## Footnotes

1
https://www.ncbi.nlm.nih.gov/

2
https://crisprcas.i2bc.paris-saclay.fr/CrisprCasFinder/Index

3
http://weblogo.berkeley.edu/logo.cgi

4
http://rna.tbi.univie.ac.at/cgi-bin/RNAWebSuite/RNAfold.cgi
