# Supplementary material for: The Clustered Regularly Interspaced Short Palindromic Repeats-Associated System and Its Relationship With Mobile Genetic Elements in Klebsiella
Source: Front Microbiol. 2022 Feb 2;12:790673. doi: 10.3389/fmicb.2021.790673 (PMC8847753; doi:10.3389/fmicb.2021.790673)
Supplement: Supplementary file 1 [file Table_1.DOCX]

Table S1

| Strain | GenBank assembly accession | Date | |
| --- | --- | --- | --- |
| *Klebsiella pneumoniae* strain KPC160121 | GCA_011106605.1 | | 2020/3/10 |
| *Klebsiella pneumoniae* strain KPC160117 | GCA_011106775.1 | | 2020/3/10 |
| *Klebsiella pneumoniae* strain KPC160125 | GCA_011106795.1 | | 2020/3/10 |
| *Klebsiella pneumoniae* strain KPC160132 | GCA_011129215.1 | | 2020/3/10 |
| *Klebsiella pneumoniae* strain Kp8701 | GCA_011068365.1 | | 2020/3/9 |
| *Klebsiella aerogenes* strain 18-2341 | GCA_011067245.1 | | 2020/3/8 |
| *Klebsiella pneumoniae* strain 16HN-263 | GCA_011066505.1 | | 2020/3/6 |
| *Klebsiella pneumoniae* strain 6 | GCA_011065345.1 | | 2020/3/5 |
| *Klebsiella pneumoniae* strain 33 | GCA_011065365.1 | | 2020/3/5 |
| *Klebsiella pneumoniae* strain 39 | GCA_011065385.1 | | 2020/3/5 |
| *Klebsiella pneumoniae* strain 2019036D | GCA_011045775.1 | | 2020/3/2 |
| *Klebsiella pneumoniae* strain QD23 | GCA_011045595.1 | | 2020/3/2 |
| *Klebsiella pneumoniae* strain 20467 | GCA_011058615.1 | | 2020/2/26 |
| *Klebsiella pneumoniae* strain KP18-2079 | GCA_011022255.1 | | 2020/2/26 |
| *Klebsiella pneumoniae* strain K2606 | GCA_011006575.1 | | 2020/2/24 |
| *Klebsiella pneumoniae* strain Xen39 | GCA_010586985.1 | | 2020/2/14 |
| *Klebsiella aerogenes* strain AUH-KAM-9 | GCA_010509815.1 | | 2020/2/13 |
| *Klebsiella pneumoniae* strain KP18-3-8 | GCA_010450855.1 | | 2020/2/12 |
| *Klebsiella pneumoniae* strain D1 | GCA_008632935.2 | | 2020/2/11 |
| *Klebsiella variicola* strain 118 | GCA_010365565.1 | | 2020/2/9 |
| *Klebsiella pneumoniae* strain C2601 | GCA_010183665.1 | | 2020/2/5 |
| *Klebsiella pneumoniae* strain C2974 | GCA_010183605.1 | | 2020/2/5 |
| *Klebsiella pneumoniae* strain C2972 | GCA_010183625.1 | | 2020/2/5 |
| *Klebsiella pneumoniae* strain C2660 | GCA_010183645.1 | | 2020/2/5 |
| *Klebsiella pneumoniae* strain C2414 | GCA_010183685.1 | | 2020/2/5 |
| *Klebsiella pneumoniae* strain C2343 | GCA_010183705.1 | | 2020/2/5 |
| *Klebsiella pneumoniae* strain C2315 | GCA_010183725.1 | | 2020/2/5 |
| *Klebsiella michiganensis* strain BD177 | GCA_010093005.1 | | 2020/2/3 |
| *Klebsiella pneumoniae* strain MRK9 | GCA_009937845.1 | | 2020/1/28 |
| *Klebsiella aerogenes* strain N1 | GCA_009937725.1 | | 2020/1/28 |
| *Klebsiella michiganensis* strain F107 | GCA_009930855.1 | | 2020/1/27 |
| *Klebsiella michiganensis* strain F107 | GCA_009930855.1 | | 2020/1/27 |
| *Klebsiella pneumoniae* strain LSH-KPN148 | GCA_009914235.1 | | 2020/1/22 |
| *Klebsiella pneumoniae* strain LSH-KPN25 | GCA_009914255.1 | | 2020/1/22 |
| *Klebsiella pneumoniae* *subsp. pneumoniae* strain KUH-KPNHVL1 | GCA_009909325.1 | | 2020/1/21 |
| *Klebsiella pneumoniae* *subsp. pneumoniae* strain KUH-KPNHVF1 | GCA_009909305.1 | | 2020/1/21 |
| *Klebsiella aerogenes* strain HNHF1 | GCA_009909445.1 | | 2020/1/21 |
| *Klebsiella pneumoniae* strain 158590 | GCA_009906875.1 | | 2020/1/20 |
| *Klebsiella pneumoniae* strain 156070 | GCA_009906895.1 | | 2020/1/20 |
| *Klebsiella pneumoniae* strain WSD411 | GCA_009884415.1 | | 2020/1/15 |
| *Klebsiella pneumoniae* strain YML0508 | GCA_009884395.1 | | 2020/1/15 |
| *Klebsiella pneumoniae* *subsp. pneumoniae* strain KP-8788 | GCA_009867115.1 | | 2020/1/14 |
| *Klebsiella pneumoniae* strain Kp36 | GCA_009856585.1 | | 2020/1/11 |
| *Klebsiella pneumoniae* kpn154 | GCA_902723695.1 | | 2020/1/11 |
| *Klebsiella pneumoniae* Kpn2166 | GCA_902723705.1 | | 2020/1/11 |
| *Klebsiella pneumoniae* strain KP19-2029 | GCA_009833945.1 | | 2020/1/8 |
| *Klebsiella pneumoniae* strain XJ-K2 | GCA_009834285.1 | | 2020/1/8 |
| *Klebsiella pneumoniae* strain BD_DM_914 | GCA_009791475.1 | | 2019/12/23 |
| *Klebsiella pneumoniae* strain BD_DM_697 | GCA_009791495.1 | | 2019/12/23 |
| *Klebsiella pneumoniae* strain WCGKP294 | GCA_009755705.1 | | 2019/12/16 |
| *Klebsiella pneumoniae* strain 2018S06-082 | GCA_009740065.1 | | 2019/12/11 |
| *Klebsiella pneumoniae* strain 2018C01-239 | GCA_009740245.1 | | 2019/12/11 |
| *Klebsiella pneumoniae* strain 2018S06-082 | GCA_009740065.1 | | 2019/12/11 |
| *Klebsiella pneumoniae* strain 2018C01-239 | GCA_009740245.1 | | 2019/12/11 |
| *Klebsiella pneumoniae* strain 2018S07-013 | GCA_009740225.1 | | 2019/12/11 |
| *Klebsiella pneumoniae* strain 2018N16-148 | GCA_009740085.1 | | 2019/12/11 |
| *Klebsiella pneumoniae* strain 2018C01-046 | GCA_009740025.1 | | 2019/12/11 |
| *Klebsiella pneumoniae* strain 2018N17-066 | GCA_009740045.1 | | 2019/12/11 |
| *Klebsiella pneumoniae* strain BD_DM_782 | GCA_009734105.1 | | 2019/12/7 |
| *Klebsiella aerogenes* strain Y6 | GCA_009732795.1 | | 2019/12/5 |
| *Klebsiella aerogenes* strain Y3 | GCA_009732815.1 | | 2019/12/5 |
| *Klebsiella aerogenes* strain Y1 | GCA_009732835.1 | | 2019/12/5 |
| *Klebsiella pneumoniae* strain KP2 | GCA_009720445.1 | | 2019/11/29 |
| *Klebsiella pneumoniae* strain C789 | GCA_009684575.1 | | 2019/11/20 |
| *Klebsiella pneumoniae* strain C1398 | GCA_009684615.1 | | 2019/11/20 |
| *Klebsiella pneumoniae* strain LEMB18 | GCA_009648995.1 | | 2019/11/11 |
| *Klebsiella pneumoniae* strain TK421 | GCA_009601745.1 | | 2019/11/6 |
| *Klebsiella pneumoniae* strain SMU18037509 | GCA_009556655.1 | | 2019/11/5 |
| *Klebsiella pneumoniae* strain 13190 | GCA_009497735.1 | | 2019/11/4 |
| *Klebsiella pneumoniae* strain K2044 | GCA_009497695.1 | | 2019/11/4 |
| *Klebsiella variicola* strain 13450 | GCA_009497715.1 | | 2019/11/4 |
| *Klebsiella pneumoniae* strain LH375 | GCA_009387915.1 | | 2019/10/29 |
| *Klebsiella pneumoniae* strain TH164 | GCA_009387815.1 | | 2019/10/29 |
| *Klebsiella pneumoniae* strain LH94 | GCA_009387775.1 | | 2019/10/29 |
| *Klebsiella quasipneumoniae* strain TH114 | GCA_009387795.1 | | 2019/10/29 |
| *Klebsiella pneumoniae* strain LH102-A | GCA_009387945.1 | | 2019/10/29 |
| *Klebsiella pneumoniae* *subsp. pneumoniae* strain BK13048 | GCA_009025895.1 | | 2019/10/14 |
| *Klebsiella michiganensis* strain C52 | GCA_008931605.1 | | 2019/10/9 |
| *Klebsiella michiganensis* strain C52 | GCA_008931605.1 | | 2019/10/9 |
| *Klebsiella pneumoniae* strain C51 | GCA_008931345.1 | | 2019/10/9 |
| *Klebsiella pneumoniae* strain C2 | GCA_008931565.1 | | 2019/10/9 |
| *Klebsiella aerogenes* strain C9 | GCA_008931665.1 | | 2019/10/9 |
| *Klebsiella grimontii* strain SS141 | GCA_004104525.2 | | 2019/10/7 |
| *Klebsiella quasipneumoniae* strain A708 | GCA_008807175.1 | | 2019/10/3 |
| *Klebsiella pneumoniae* strain KP65 | GCA_008728415.1 | | 2019/9/30 |
| *Klebsiella pneumoniae* strain KP58 | GCA_008728695.1 | | 2019/9/30 |
| *Klebsiella aerogenes* strain KA_P10_L5_03.19 | GCA_008727695.1 | | 2019/9/29 |
| *Klebsiella pneumoniae* strain FDAARGOS_630 | GCA_008693405.1 | | 2019/9/25 |
| *Klebsiella pneumoniae* strain RJY9645 | GCA_006965405.3 | | 2019/9/25 |
| *Klebsiella pneumoniae* strain FDAARGOS_631 | GCA_008693425.1 | | 2019/9/25 |
| *Klebsiella pneumoniae* strain FDAARGOS_629 | GCA_008693445.1 | | 2019/9/25 |
| *Klebsiella variicola* strain FDAARGOS_627 | GCA_008693465.1 | | 2019/9/25 |
| *Klebsiella michiganensis* strain FDAARGOS_647 | GCA_008693565.1 | | 2019/9/25 |
| *Klebsiella aerogenes* strain FDAARGOS_641 | GCA_008693885.1 | | 2019/9/25 |
| *Klebsiella pneumoniae* strain 555 | GCA_008632415.1 | | 2019/9/22 |
| *Klebsiella pneumoniae* strain 555 | GCA_008632415.1 | | 2019/9/22 |
| *Klebsiella quasipneumoniae* subsp. quasipneumoniae strain M17277 | GCA_008632435.1 | | 2019/9/22 |
| *Klebsiella pneumoniae* strain RJ18-01 | GCA_008386275.1 | | 2019/9/16 |
| *Klebsiella pneumoniae* strain P094-1 | GCA_008369685.1 | | 2019/9/11 |
| *Klebsiella quasipneumoniae* SNI47 | GCA_009002435.1 | | 2019/9/9 |
| *Klebsiella pneumoniae* strain KLP268 | GCA_008121515.1 | | 2019/8/28 |
| *Klebsiella pneumoniae* strain KP1692 | GCA_008122305.1 | | 2019/8/28 |
| *Klebsiella pneumoniae* strain KP1677 | GCA_008122285.1 | | 2019/8/28 |
| *Klebsiella pneumoniae* strain 18-2374 | GCA_008065415.1 | | 2019/8/22 |
| *Klebsiella pneumoniae* strain PIMB15ND2KP27 | GCA_007873865.1 | | 2019/8/7 |
| *Klebsiella pneumoniae* strain DA12090 | GCA_007833575.1 | | 2019/8/5 |
| *Klebsiella pneumoniae* strain IA565 | GCA_007833555.1 | | 2019/8/5 |
| *Klebsiella pneumoniae* strain KP14003 | GCA_003429065.3 | | 2019/7/31 |
| *Klebsiella pneumoniae* strain KP14003 | GCA_003429065.3 | | 2019/7/31 |
| *Klebsiella aerogenes* strain Ka37751 | GCA_007632255.1 | | 2019/7/30 |
| *Klebsiella pneumoniae* strain NKU_KlebA1 | GCA_007999005.1 | | 2019/7/23 |
| *Klebsiella pneumoniae* strain NKU_Kleb8A7 | GCA_007197815.1 | | 2019/7/21 |
| *Klebsiella michiganensis* strain KNU07 | GCA_007106885.1 | | 2019/7/18 |
| *Klebsiella pneumoniae* strain R50 | GCA_006974165.1 | | 2019/7/15 |
| *Klebsiella aerogenes* strain LU2 | GCA_006874725.1 | | 2019/7/11 |
| *Klebsiella pneumoniae* strain L201 | GCA_003111805.2 | | 2019/7/11 |
| *Klebsiella pneumoniae* strain L491 | GCA_003111885.2 | | 2019/7/11 |
| *Klebsiella pneumoniae* strain L388 | GCA_003112125.1 | | 2019/7/11 |
| *Klebsiella aerogenes* strain 4928STDY7071344 | GCA_902164615.1 | | 2019/7/9 |
| *Klebsiella pneumoniae* strain 4928STDY7071295 | GCA_902164625.1 | | 2019/7/9 |
| *Klebsiella pneumoniae* strain 4928STDY7071681 | GCA_902166475.1 | | 2019/7/9 |
| *Klebsiella pneumoniae* strain 4928STDY7071680 | GCA_902166495.1 | | 2019/7/9 |
| *Klebsiella pneumoniae* strain 4928STDY7071137 | GCA_902166515.1 | | 2019/7/9 |
| *Klebsiella pneumoniae* strain 4928STDY7387729 | GCA_902166745.1 | | 2019/7/9 |
| *Klebsiella pneumoniae* strain L39_2 | GCA_006494895.1 | | 2019/6/27 |
| *Klebsiella pneumoniae* strain L482 | GCA_006494915.1 | | 2019/6/27 |
| *Klebsiella pneumoniae* strain Kp202 | GCA_006459145.1_ | | 2019/6/25 |
| *Klebsiella pneumoniae* strain FDAARGOS_775 | GCA_006364295.1 | | 2019/6/19 |
| *Klebsiella oxytoca* strain NCTC13727 | GCA_006364295.1 | | 2019/6/19 |
| *Klebsiella pneumoniae* strain XJ-K1 | GCA_006304565.1 | | 2019/6/12 |
| *Klebsiella quasipneumoniae* isolate SGM81 | GCA_900187335.1 | | 2019/6/9 |
| *Klebsiella pneumoniae* strain KpvST147B_SE1_1_NDM | GCA_005944305.1 | | 2019/6/4 |
| *Klebsiella pneumoniae* *subsp. pneumoniae* strain KpvST15_NDM | GCA_005885775.1 | | 2019/5/29 |
| *Klebsiella pneumoniae* strain CR-HvKP1 | GCA_005853785.1 | | 2019/5/27 |
| *Klebsiella pneumoniae* strain CR-HvKP5 | GCA_005854025.1 | | 2019/5/27 |
| *Klebsiella pneumoniae* strain CR-HvKP4 | GCA_005854245.1 | | 2019/5/27 |
| *Klebsiella pneumoniae* strain KpvST101_OXA-48 | GCA_003363735.2 | | 2019/5/22 |
| *Klebsiella pneumoniae* strain KpvST101_OXA-48 | GCA_003363735.2 | | 2019/5/22 |
| *Klebsiella pneumoniae* *subsp. pneumoniae* KpvST383_NDM_OXA-48 | GCA_003860645.2 | | 2019/5/22 |
| *Klebsiella pneumoniae* strain 2e | GCA_005377825.1 | | 2019/5/15 |
| *Klebsiella pneumoniae* strain CRKP I | GCA_005347525.1 | | 2019/5/14 |
| *Klebsiella pneumoniae* strain NCTC9171 | GCA_901421965.1 | | 2019/5/9 |
| *Klebsiella pneumoniae* strain NCTC9157 | GCA_901422065.1 | | 2019/5/9 |
| *Klebsiella pneumoniae* strain R1701 | GCA_005239265.1 | | 2019/5/8 |
| *Klebsiella pneumoniae* strain R1761 | GCA_005239285.1 | | 2019/5/8 |
| *Klebsiella pneumoniae* strain ST23 | GCA_004924315.1 | | 2019/4/30 |
| *Klebsiella pneumoniae* TA6363 | GCA_010367305.1 | | 2019/4/26 |
| *Klebsiella pneumoniae* strain 121 | GCA_004801235.1 | | 2019/4/19 |
| *Klebsiella pneumoniae* strain 18CPO060 | GCA_004332075.1 | | 2019/3/7 |
| *Klebsiella pneumoniae* strain ABFQB | GCA_004322995.1 | | 2019/3/4 |
| *Klebsiella pneumoniae* strain_ABFPV | GCA_004322955.1 | | 2019/3/4 |
| *Klebsiella pneumoniae* strain WCHKP115068 | GCA_004322795.1 | | 2019/3/3 |
| *Klebsiella pneumoniae* strain BP327 | GCA_004319525.1 | | 2019/2/28 |
| *Klebsiella pneumoniae* strain BA34918 | GCA_004295405.1 | | 2019/2/25 |
| *Klebsiella pneumoniae* strain BA1559 | GCA_004295385.1 | | 2019/2/25 |
| Klebsiella huaxiensis strain WCHKl090001 | GCA_003261575.2 | | 2019/2/25 |
| *Klebsiella pneumoniae* strain BA4656 | GCA_004209795.1 | | 2019/2/19 |
| *Klebsiella pneumoniae* strain R46 | GCA_004208475.1 | | 2019/2/16 |
| *Klebsiella pneumoniae* *subsp. pneumoniae* strain CCRI-22199 | GCA_004141975.1 | | 2019/2/6 |
| *Klebsiella pneumoniae* strain 2-1 | GCA_004138665.1 | | 2019/2/5 |
| *Klebsiella pneumoniae* strain NFYY0065 | GCA_004137665.1 | | 2019/2/5 |
| *Klebsiella pneumoniae* strain NH34 | GCA_004135795.1 | | 2019/2/4 |
| *Klebsiella pneumoniae* strain AP8555 | GCA_004118955.1 | | 2019/1/30 |
| *Klebsiella pneumoniae* strain F5 | GCA_004119955.1 | | 2019/1/30 |
| *Klebsiella pneumoniae* strain F77 | GCA_004119975.1 | | 2019/1/30 |
| *Klebsiella pneumoniae* strain F10 | GCA_004120015.1 | | 2019/1/30 |
| *Klebsiella pneumoniae* strain F93-1 | GCA_004120035.1 | | 2019/1/30 |
| *Klebsiella pneumoniae* strain F13 | GCA_004120055.1 | | 2019/1/30 |
| *Klebsiella pneumoniae* strain F132 | GCA_004120075.1 | | 2019/1/30 |
| *Klebsiella pneumoniae* strain F138 | GCA_004120095.1 | | 2019/1/30 |
| *Klebsiella pneumoniae* strain B12 | GCA_004120115.1 | | 2019/1/30 |
| *Klebsiella pneumoniae* strain F81 | GCA_004120155.1 | | 2019/1/30 |
| *Klebsiella pneumoniae* strain F93-2 | GCA_004120175.1 | | 2019/1/30 |
| *Klebsiella pneumoniae* strain F89-1 | GCA_004120135.1 | | 2019/1/30 |
| *Klebsiella pneumoniae* isolate KP980 | GCA_900497045.1 | | 2019/1/29 |
| *Klebsiella pneumoniae* isolate KP9201 | GCA_900497055.1 | | 2019/1/29 |
| *Klebsiella michiganensis* strain M82255 | GCA_004102625.1 | | 2019/1/22 |
| *Klebsiella variicola* strain 15WZ-82 | GCA_004014895.1 | | 2019/1/14 |
| *Klebsiella variicola* strain 15WZ-82 | GCA_004014895.1 | | 2019/1/14 |
| *Klebsiella sp.* LY | GCA_004010915.1 | | 2019/1/10 |
| *Klebsiella pneumoniae* strain 08EU827 | GCA_004010995.1 | | 2019/1/10 |
| *Klebsiella pneumoniae* strain L5-2 | GCA_004014965.1 | | 2019/1/10 |
| *Klebsiella variicola* strain AJ055 | GCA_900622585.1 | | 2019/1/10 |
| *Klebsiella pneumoniae* strain AJ218 | GCA_900622605.1 | | 2019/1/10 |
| *Klebsiella pneumoniae* strain KPC2 | GCA_900622645.1 | | 2019/1/10 |
| *Klebsiella variicola* strain AJ292 | GCA_900622595.1 | | 2019/1/10 |
| *Klebsiella variicola* strain 04153260899A | GCA_900622615.1 | | 2019/1/10 |
| *Klebsiella variicola* strain 03-311-007 | GCA_900622625.1 | | 2019/1/10 |
| *Klebsiella pneumoniae* strain NB5306 | GCA_004006035.1 | | 2019/1/9 |
| *Klebsiella quasipneumoniae* strain D120-1 | GCA_003990375.1 | | 2019/1/2 |
| *Klebsiella quasipneumoniae* strain D120-1 | GCA_003990375.1 | | 2019/1/2 |
| *Klebsiella pneumoniae* strain T4 | GCA_003963495.1 | | 2018/12/23 |
| *Klebsiella aerogenes* strain NCTC9644 | GCA_900636315.1 | | 2018/12/20 |
| *Klebsiella aerogenes* strain NCTC9735 | GCA_900637945.1 | | 2018/12/20 |
| *Klebsiella pneumoniae* strain TOP52_1721_U1 | GCA_003957455.1 | | 2018/12/20 |
| *Klebsiella aerogenes* strain NCTC10006 | GCA_900635435.1 | | 2018/12/19 |
| *Klebsiella oxytoca* strain NCTC11356 | GCA_900635105.1 | | 2018/12/19 |
| *Klebsiella pneumoniae* strain NCTC11359 | GCA_900635125.1 | | 2018/12/19 |
| *Klebsiella pneumoniae* strain NCTC13635 | GCA_900635735.1 | | 2018/12/19 |
| *Klebsiella aerogenes* strain NCTC9652 | GCA_900636095.1 | | 2018/12/19 |
| *Klebsiella pneumoniae* strain 11492 | GCA_003953905.1 | | 2018/12/18 |
| *Klebsiella pneumoniae* isolate KSH203 | GCA_003940845.1 | | 2018/12/12 |
| *Klebsiella pneumoniae* strain I72 | GCA_003934185.1 | | 2018/12/11 |
| *Klebsiella pneumoniae* strain cr-hvkp3 | GCA_003932915.1 | | 2018/12/10 |
| *Klebsiella pneumoniae* strain KP18-29 | GCA_003931835.1 | | 2018/12/9 |
| *Klebsiella pneumoniae* strain 4/1-2 | GCA_003856595.1 | | 2018/12/3 |
| *Klebsiella pneumoniae* strain BJCFK909 | GCA_003856475.1 | | 2018/12/3 |
| *Klebsiella pneumoniae* strain KP_NORM_BLD_2014_104014 | GCA_003855315.1 | | 2018/12/2 |
| *Klebsiella pneumoniae* strain KP_NORM_BLD_2015_112126 | GCA_003855335.1 | | 2018/12/2 |
| *Klebsiella pneumoniae* *subsp. pneumoniae* strain R210-2 | GCA_003855515.1 | | 2018/12/2 |
| *Klebsiella pneumoniae* *subsp. pneumoniae* strain ARLG-3135 | GCA_003850165.1 | | 2018/11/29 |
| *Klebsiella pneumoniae* strain KPN1343 | GCA_003815075.1 | | 2018/11/24 |
| *Klebsiella pneumoniae* strain KPN1344 | GCA_003815095.1 | | 2018/11/24 |
| *Klebsiella oxytoca* strain FDAARGOS_500 | GCA_003812925.1 | | 2018/11/21 |
| *Klebsiella pneumoniae* strain FDAARGOS_566 | GCA_003812085.1 | | 2018/11/21 |
| *Klebsiella pneumoniae* strain FDAARGOS_531 | GCA_003812105.1 | | 2018/11/21 |
| *Klebsiella aerogenes* strain FDAARGOS_513 | GCA_003812185.1 | | 2018/11/21 |
| Klebsiella sp. FDAARGOS_511 | GCA_003812845.1 | | 2018/11/21 |
| *Klebsiella pneumoniae* strain 4743 | GCA_003790485.1 | | 2018/11/19 |
| *Klebsiella pneumoniae* strain WCHKP015625 | GCA_003261955.2 | | 2018/11/5 |
| *Klebsiella pneumoniae* strain WCHKP115069 | GCA_003711065.1 | | 2018/11/5 |
| *Klebsiella pneumoniae* strain 675920 | GCA_003703775.1 | | 2018/10/31 |
| *Klebsiella pneumoniae* strain 2e | GCA_003692695.1 | | 2018/10/29 |
| *Klebsiella pneumoniae* strain INF237 | GCA_003660205.1 | | 2018/10/16 |
| *Klebsiella pneumoniae* strain INF078 | GCA_003660185.1 | | 2018/10/16 |
| *Klebsiella pneumoniae* strain ST307PT04 | GCA_003597695.1 | | 2018/9/26 |
| *Klebsiella pneumoniae* strain ST307PT02 | GCA_003597715.1 | | 2018/9/26 |
| *Klebsiella pneumoniae* strain ST307PT03 | GCA_003597735.1 | | 2018/9/26 |
| *Klebsiella pneumoniae* strain ST307PT01 | GCA_003597755.1 | | 2018/9/26 |
| *Klebsiella pneumoniae* strain INF235-sc-2280127 | GCA_003590665.1 | | 2018/9/24 |
| *Klebsiella pneumoniae* strain AR_0046 | GCA_003571745.1 | | 2018/9/16 |
| *Klebsiella pneumoniae* strain AR_0076 | GCA_003571525.1 | | 2018/9/16 |
| *Klebsiella pneumoniae* strain AR_0135 | GCA_003571545.1 | | 2018/9/16 |
| *Klebsiella pneumoniae* strain AR_0160 | GCA_003571585.1 | | 2018/9/16 |
| *Klebsiella pneumoniae* strain AR_0075 | GCA_003571605.1 | | 2018/9/16 |
| *Klebsiella pneumoniae* strain AR_0097 | GCA_003571645.1 | | 2018/9/16 |
| *Klebsiella pneumoniae* strain AR_0109 | GCA_003571705.1 | | 2018/9/16 |
| *Klebsiella aerogenes* strain FDAARGOS_327 | GCA_003546885.1 | | 2018/9/11 |
| *Klebsiella pneumoniae* strain 1050 | GCA_003443755.1 | | 2018/9/4 |
| *Klebsiella quasipneumoniae* strain L22 | GCA_003443835.1 | | 2018/9/4 |
| *Klebsiella pneumoniae* strain INF125-sc-2279943 | GCA_003432285.1 | | 2018/8/30 |
| *Klebsiella pneumoniae* strain KSB1_1I-sc-2280289 | GCA_003432145.1 | | 2018/8/30 |
| *Klebsiella pneumoniae* strain MSB1_8A-sc-2280397 | GCA_003432165.1 | | 2018/8/30 |
| *Klebsiella pneumoniae* strain INF206-sc-2280074 | GCA_003432185.1 | | 2018/8/30 |
| *Klebsiella pneumoniae* strain KSB1_7F-sc-2280268 | GCA_003432205.1 | | 2018/8/30 |
| *Klebsiella pneumoniae* strain INF014-sc-2279884 | GCA_003432225.1 | | 2018/8/30 |
| *Klebsiella pneumoniae* strain INF116-sc-2279924 | GCA_003432245.1 | | 2018/8/30 |
| *Klebsiella pneumoniae* strain QMP B2-170 | GCA_003432265.1 | | 2018/8/30 |
| *Klebsiella pneumoniae* strain ZYST1 | GCA_003432405.1 | | 2018/8/30 |
| *Klebsiella pneumoniae* strain 160111 | GCA_003429545.1 | | 2018/8/28 |
| *Klebsiella variicola* strain X39 | GCA_003429625.1 | | 2018/8/28 |
| *Klebsiella pneumoniae* *subsp. pneumoniae* strain M5 | GCA_003428845.1 | | 2018/8/27 |
| *Klebsiella pneumoniae* strain N4b | GCA_003408615.1 | | 2018/8/20 |
| *Klebsiella pneumoniae* strain N4b | GCA_003408615.1 | | 2018/8/20 |
| *Klebsiella pneumoniae* *subsp. pneumoniae* strain JNM8C2 | GCA_003368345.1 | | 2018/8/9 |
| *Klebsiella pneumoniae* strain JNM10C3 | GCA_003368365.1 | | 2018/8/9 |
| *Klebsiella pneumoniae* *subsp. pneumoniae* strain JNM8C2 | GCA_003368345.1 | | 2018/8/9 |
| *Klebsiella pneumoniae* strain JNM10C3 | GCA_003368365.1 | | 2018/8/9 |
| *Klebsiella pneumoniae* strain KP30835 | GCA_003368025.1 | | 2018/8/9 |
| *Klebsiella pneumoniae* strain F1 | GCA_003367555.1 | | 2018/8/8 |
| *Klebsiella pneumoniae* KP33 DNA | GCA_003574235.1 | | 2018/7/20 |
| *Klebsiella pneumoniae* KP64 | GCA_003574255.1 | | 2018/7/20 |
| *Klebsiella pneumoniae* KP67 | GCA_003574275.1 | | 2018/7/20 |
| *Klebsiella pneumoniae* *subsp. pneumoniae* strain KC-Pl-HB1 | GCA_003330945.1 | | 2018/7/20 |
| *Klebsiella variicola* strain E57-7 | GCA_003290425.1 | | 2018/7/9 |
| *Klebsiella pneumoniae* strain AR_362 | GCA_003288375.1 | | 2018/7/5 |
| *Klebsiella pneumoniae* *subsp. pneumoniae* strain SC-7 | GCA_003286995.1 | | 2018/7/3 |
| *Klebsiella pneumoniae* isolate CNR48 | GCA_900323945.1 | | 2018/7/3 |
| *Klebsiella pneumoniae* *subsp. pneumoniae* strain 12208 | GCA_003286975.1 | | 2018/7/3 |
| *Klebsiella quasipneumoniae* subsp. quasipneumoniae strain A708 | GCA_003285165.1 | | 2018/7/2 |
| *Klebsiella variicola* strain 13450 | GCA_003285185.1 | | 2018/7/2 |
| *Klebsiella pneumoniae* CDC 0106 | GCA_003267985.1 | | 2018/6/26 |
| *Klebsiella oxytoca* strain NCTC11355 | GCA_900478285.1 | | 2018/6/18 |
| *Klebsiella michiganensis* strain AACKY | GCA_003205795.1 | | 2018/6/11 |
| *Klebsiella pneumoniae* strain AR_0140 | GCA_003204465.1 | | 2018/6/10 |
| *Klebsiella pneumoniae* strain AR_0087 | GCA_003204175.1 | | 2018/6/10 |
| *Klebsiella pneumoniae* strain TVGHCRE225 | GCA_003203435.1 | | 2018/6/8 |
| *Klebsiella pneumoniae* strain TVGHCRE225 | GCA_003203435.1 | | 2018/6/8 |
| *Klebsiella pneumoniae* strain TVGHCRE225 | GCA_003203435.1 | | 2018/6/8 |
| *Klebsiella pneumoniae* strain CCUG 70742 | GCA_003194285.1 | | 2018/6/6 |
| *Klebsiella pneumoniae* strain CCUG 70747 | GCA_003194325.1 | | 2018/6/6 |
| *Klebsiella pneumoniae* strain 203 | GCA_003186535.1 | | 2018/6/5 |
| *Klebsiella pneumoniae* GSU10-3 | GCA_003584585.1 | | 2018/6/5 |
| *Klebsiella pneumoniae* strain DA33140 | GCA_003181055.1 | | 2018/5/31 |
| *Klebsiella pneumoniae* strain DA33141 | GCA_003181095.1 | | 2018/5/31 |
| *Klebsiella pneumoniae* strain DA33144 | GCA_003181135.1 | | 2018/5/31 |
| *Klebsiella quasipneumoniae* subsp. similipneumoniae strain ATCC 700603 | GCA_003181175.1 | | 2018/5/31 |
| *Klebsiella quasipneumoniae* strain CAV2018 | GCA_003146635.1 | | 2018/5/22 |
| *Klebsiella quasipneumoniae* strain CAV2013 | GCA_003146655.1 | | 2018/5/22 |
| *Klebsiella quasipneumoniae* strain CAV1947 | GCA_003146685.1 | | 2018/5/22 |
| *Klebsiella pneumoniae* strain NCTC 418 | GCA_003076915.1 | | 2018/5/2 |
| *Klebsiella pneumoniae* strain 616 | GCA_003076555.1 | | 2018/5/1 |
| *Klebsiella pneumoniae* strain AR376 | GCA_003074015.1 | | 2018/4/30 |
| *Klebsiella michiganensis* strain AR375 | GCA_003074075.1 | | 2018/4/30 |

Table S2

| Plasmid | GenBank sequence |
| --- | --- |
| p2018C01-239-1_MCR1 | CP044386.1 |
| p2018S06-082-1_MCR1 | CP044377.1 |
| pSCKLB555-1 | CP043933.1 |
| p15WZ-82_Vir, | CP032356.1 |
| pD120-1_296kb | CP034681.1 |
| plasmid unnamed1 | CP023723.1 |
| pKpvST101_5 | CP031372.2 |
| pKJNM8C2.1 | CP030858.1 |
| pKJNM10C3.2 | CP030878.1 |
| p1502320-3 | CP031580.1 |
| plasmid unnamed1 | CP041935.1 |
| p2018S07-013-1 | CP044381.1 |
| p2018N16-148-2_MCR8 | CP044395.1 |
| p2018C01-046-1_MCR8 | CP044369.1 |
| pKpvST101 | CP031369.2 |
| pKJNM8C2.2 | CP030859.1 |
| pKJNM10C3.1 | CP030876.1 |
| pDA12090.1 | CP030071.1 |
| pC2972-4-KPC | CP039798.1 |
| Plasmid pIncHIB | CP036336.1 |

Table S3

| **Query Plasmid** | **Subject Plasmid** | **Query Cover** |
| --- | --- | --- |
| plasmid pIncHIB | plasmid unnamed1-tv | 65% |
| plasmid pIncHIB | plasmid unnamed1-tv | 65% |
| plasmid pIncHIB | plasmid p1502320-3 | 76% |
| plasmid pIncHIB | plasmid pKJNM10C3.2 | 72% |
| plasmid pIncHIB | plasmid pKJNM8C2.1 | 67% |
| plasmid pIncHIB | plasmid pKpvST101_5 | 83% |
| plasmid pIncHIB | plasmid unnamed1 | 52% |
| plasmid pIncHIB | plasmid pD120-1_296kb | 52% |
| plasmid pIncHIB | plasmid p15WZ-82_Vir | 52% |
| plasmid pIncHIB | pSCKLB555-1 | 73% |
| plasmid pIncHIB | p2018S06-082-1_MCR1 | 59% |
| plasmid pIncHIB | p2018C01-239-1_MCR1 | 60% |
| p2018C01-239-1_MCR1 | p2018S06-082-1_MCR1 | 97% |
| p2018C01-239-1_MCR1 | plasmid pKpvST101_5 | 58% |
| p2018C01-239-1_MCR1 | pSCKLB555-1 | 70% |
| p2018C01-239-1_MCR1 | plasmid unnamed1 | 76% |
| p2018C01-239-1_MCR1 | plasmid pKJNM8C2.1 | 68% |
| p2018C01-239-1_MCR1 | plasmid pD120-1_296kb | 64% |
| p2018C01-239-1_MCR1 | plasmid unnamed1-tv | 60% |
| p2018C01-239-1_MCR1 | plasmid pKJNM10C3.2 | 70% |
| p2018C01-239-1_MCR1 | plasmid p1502320-3 | 26% |
| p2018C01-239-1_MCR1 | plasmid p15WZ-82_Vir | 59% |
| p2018S06-082-1_MCR1 | plasmid pKpvST101_5 | 57% |
| p2018S06-082-1_MCR1 | pSCKLB555-1 | 69% |
| p2018S06-082-1_MCR1 | plasmid unnamed1 | 75% |
| p2018S06-082-1_MCR1 | plasmid pKJNM8C2.1 | 67% |
| p2018S06-082-1_MCR1 | plasmid pD120-1_296kb | 63% |
| p2018S06-082-1_MCR1 | plasmid unnamed1-tv | 59% |
| p2018S06-082-1_MCR1 | plasmid pKJNM10C3.2 | 70% |
| p2018S06-082-1_MCR1 | plasmid p1502320-3 | 26% |
| p2018S06-082-1_MCR1 | plasmid p15WZ-82_Vir | 57% |
| p15WZ-82_Vir | plasmid unnamed1-tv | 89% |
| p15WZ-82_Vir | plasmid pD120-1_296kb | 65% |
| p15WZ-82_Vir | pSCKLB555-1 | 65% |
| p15WZ-82_Vir | pKJNM10C3.2 | 67% |
| p15WZ-82_Vir | plasmid unnamed1 | 68% |
| p15WZ-82_Vir | pKJNM8C2.1 | 61% |
| p15WZ-82_Vir | pKpvST101_5 | 58% |
| p15WZ-82_Vir | p1502320-3 | 28% |
| p1502320-3 | plasmid unnamed1 | 95% |
| p1502320-3 | pKJNM10C3.2 | 97% |
| p1502320-3 | pKJNM8C2.1 | 90% |
| p1502320-3 | pSCKLB555-1 | 89% |
| p1502320-3 | pD120-1_296kb | 88% |
| p1502320-3 | plasmid unnamed1-tv | 82% |
| p1502320-3 | pKpvST101_5 | 62% |
| pD120-1_296kb | plasmid unnamed1-tv | 66% |
| pD120-1_296kb | pSCKLB555-1 | 67% |
| pD120-1_296kb | plasmid unnamed1 | 68% |
| pD120-1_296kb | pKJNM10C3.2 | 66% |
| pD120-1_296kb | pKJNM8C2.1 | 64% |
| pD120-1_296kb | pKpvST101_5 | 57% |
| pKJNM8C2.1 | plasmid unnamed1 | 71% |
| pKJNM8C2.1 | pSCKLB555-1 | 70% |
| pKJNM8C2.1 | plasmid unnamed1-tv | 58% |
| pKJNM8C2.1 | pKJNM10C3.2 | 86% |
| pKJNM8C2.1 | pKpvST101_5 | 61% |
| pKJNM10C3.2 | plasmid unnamed1-tv | 69% |
| pKJNM10C3.2 | pSCKLB555-1 | 80% |
| pKJNM10C3.2 | plasmid unnamed1 | 80% |
| pKJNM10C3.2 | pKJNM8C2.1 | 85% |
| pKpvST101_5 | pSCKLB555-1 | 85% |
| pKpvST101_5 | plasmid unnamed1 | 85% |
| pKpvST101_5 | plasmid unnamed1-tv | 84% |
| plasmid unnamed1-tv | pSCKLB555-1 | 64% |
| plasmid unnamed1-tv | plasmid unnamed1 | 66% |
| plasmid unnamed1 | pSCKLB555-1 | 72% |
